# Euglycemic Diabetic Ketoacidosis after Discontinuing SGLT2 Inhibitor

**DOI:** 10.1155/2022/4101975

**Published:** 2022-03-02

**Authors:** Mohamed Alhemeiri, Eiman Alseddeeqi

**Affiliations:** ^1^Medical Education Department, Sheikh Khalifa Medical City, P.O. Box 51900, Abu Dhabi, UAE; ^2^Division of Endocrinology, Sheikh Khalifa Medical City, P.O. Box51900, Abu Dhabi, UAE

## Abstract

**Background:**

Sodium glucose cotransporter-2 (SGLT2) inhibitors have been proven to be very effective in the management of type II diabetes. These medications can cause adverse drug reactions such as genital mycotic infections. Another critical adverse drug reaction is euglycemic diabetic ketoacidosis (EDKA) under the setting of other contributing risk factors for developing diabetic ketoacidosis. *Case Presentation*. We report a case of a 45-year-old gentleman with type 2 diabetes mellitus on empagliflozin, metformin, and glimepiride who presented with abdominal pain, fatigue, and vomiting. Of note, he started a ketogenic diet three days before his presentation and self-stopped his antidiabetic medications two days before his presentation. The patient was found to have euglycemic diabetic ketoacidosis and was treated as per the protocol. He was discharged on metformin and pioglitazone. Two weeks following discharge, canagliflozin was added.

**Conclusion:**

Euglycemic diabetic ketoacidosis could still be precipitated despite discontinuation of SGLT2I under a ketogenic diet. Discussion related to the initiation of a ketogenic diet should occur between the care provider and the patient.

## 1. Background

Euglycemic diabetic ketoacidosis (EDKA) is defined by a blood glucose level that is below 200 mg/dl (11.1 mmol/L), metabolic acidosis, and ketosis [1]. Multiple risk factors may predispose a patient to have EDKA, such as heavy alcohol consumption, chronic liver disease, glycogen storage disease, decreased calorie intake, infections, and dehydration. Other factors that are encountered in the in-patient setting are sepsis, major surgeries, and cerebrovascular events. Pregnancy may also cause EDKA as it is a state of physiological hypoinsulinemia [2–4]. The prognosis is usually excellent if the condition is recognized early and treated appropriately [4].

Sodium glucose cotransporter-2 (SGLT2) inhibitors lower blood glucose by a mechanism which prevents glucose reabsorption in the proximal tubule of the kidney, thus increasing urinary excretion of glucose [5]. Empagliflozin, one of the SGLT2 inhibitors, reaches a peak plasma concentration 1.33–3.0 hours after administration, half-life concentration ranged between 5.6–13.1 hours, and the trough concentration remains constant after day 6 [6]. Other studies have shown that the pharmacological effects of SGLT2 inhibitors may persist for more than 10 days as evident by the presence of glucosuria after stopping the medication for 10 days [7].

Multiple side effects are known to be caused by SGLT2 inhibitors. The most common adverse effect is an increased risk of genital mycotic infection; however, a rare but serious side effect is diabetic ketoacidosis [8]. About 2.6%–3.2% of diabetic ketoacidosis admissions are found to be euglycemic [4]. DKA associated with the use of SGLT2 inhibitor was found to be 0.16–0.76 events per 1000 patient-years in patients who are diagnosed with type 2 diabetes [4].

A causality assessment between SGLT2 and urinary tract infection and diabetic ketoacidosis was done and concluded that there is a correlation between SGLT2 and these two adverse effects [9]. Clinical trials are still ongoing to assess risk of diabetic ketoacidosis and bone fractures in patients who are using SGLT2 inhibitors [10]. A proposed mechanism on how SGLT2 causes DKA is by the fact that they cause a decreased amount of glucose in the blood via the mechanism of action which involves binding to the proximal tubule of the kidney and preventing the reabsorption of glucose to the blood [10]. The lower amount of glucose in the blood can increase lipolysis which increases the amount of free fatty acids and beta-oxidation, thus producing ketones [10].

Empagliflozin, dapagliflozin, and canagliflozin have all been associated to cause euglycemic diabetic ketoacidosis as well [1, 11, 12]. Most cases that describe EDKA in patients who are taking SGLT2 inhibitors discuss it in patients who have been on SGLT2 for a period of time who then started a ketogenic diet for one week or more before developing EDKA during their treatment course [13–16]. A ketogenic diet is defined as a diet where there is significant decrease in the amount of carbohydrates consumed typically to less than 50 g per day with an increase in proportion of fat and protein consumed to very high percentages [17].

Our case is unusual because it is the first one describing a EDKA status in a patient who was off SGLT2 inhibitors for two days while starting a ketogenic diet for three days duration. The case is of importance to the medical literature as it discusses a potential and serious side effect of SGLT2 inhibitors even after stopping the medication. It sheds light on the need for a discussion between the care provider and the patient regarding whether the patient is a candidate for and when to initiate a ketogenic diet.

## 2. Case Report

A 45-year-old gentleman of an Arabic ethnicity, with type 2 diabetes mellitus, hypertension, and dyslipidemia for 10 years duration and well controlled asthma presented to the emergency department complaining of abdominal pain, fatigue, nausea, and vomiting for 1 day. He denies fever, cough, chest pain, and diarrhea. Antihyperglycemic medications at the time of arrival included glimepiride, empagliflozin 25 mg once daily, and metformin 1,000 mg once daily. Other medications include telmisartan 80 mg once daily, amlodipine 5 mg once daily, atorvastatin 20 mg once daily, and salbutamol 100 mcg/actuation as needed for asthma exacerbations. He has no allergies to medications and there is no significant family history. He is an active smoker with a 30 pack-year history.

Three days before his presentation, he started a keto diet which included a carbohydrate amount of 30 grams only per day. He self-stopped all of his antidiabetic medications two days before his presentation. On examination, the patient was afebrile and tachycardic. The patient's weight was 115 kg and height was 178 cm which constitute a body mass index of 36.3 kg/m^2^. Abdominal examination revealed mild epigastric tenderness.

Laboratory values upon presentation to the emergency department were as follows: glucose 135 mg/dl (70–110 mg/dl)/7.5 mmol/l (3.9–6.1 mmol/L), HbA1c 9.7% (4.4–5.6%), sodium 132 mmol/L (136–145 mmol/L), chloride 97 mmol/L (98–107 mmol/L), venous bicarbonate 6 mmol/L (22–26 mmol/L), anion gap 29 [6–8, 11–16], urinary ketones 15 (3+) mmol/l, and urinary glucose 56 (4+) mmol/L. All lab values were taken from venous samples (Table 1). The patient was diagnosed with euglycemic DKA and was admitted to the medical ward and treatment with dextrose and insulin drips was initiated as per the hospital DKA protocol. During the hospital stay, the serum lactic acid level was 0.8 mmol/L and serum beta-hydroxybutyric acid level was 4.21 mmol/L.

The patient was discharged after being hospitalized for four days. The discharge medications included metformin 1g BID and pioglitazone 15 mg BID. The patient presented for follow-up to the endocrinology clinic after 2 weeks where canagliflozin 300 mg was added.

## 3. Discussion

We report a case of euglycemic diabetic ketoacidosis in a patient who was on a ketogenic diet. To the best of our knowledge, this is the first case of EDKA in a patient who stopped his SGLT2 inhibitor while starting a ketogenic diet and yet developed EDKA.

Diabetic ketoacidosis is defined by the triad of hyperglycemia, ketones, and raised anion gap metabolic acidosis. Euglycemic diabetic ketoacidosis is characterized by milder blood glucose levels which are below 200 mg/dl (11.1 mmol/L) along with an anion gap metabolic acidosis and ketonemia or ketonuria [18]. Overall, 2.6%–3.2% of diabetic ketoacidosis admissions are found to be euglycemic [4].

SGLT2 inhibitors are known to be predisposed to DKA and have even received a warning by the Food and Drug Administration in May 2015 warning about this side effect [19]. Studies have shown multiple mechanisms as to why DKA may occur in the use of SGLT2 inhibitors. One of the hypothesized reasons is that empagliflozin and dapagliflozin may increase the levels of glucagon via action on the pancreatic alpha cell, thus promoting hepatic ketogenesis and driving ketone formation [19]. However, not all patients who are on SGLT2 inhibitors might develop euglycemic DKA. Other risk factors for developing euglycemic DKA include recent use of insulin, pregnancy, heavy alcohol consumption, chronic liver disease, glycogen storage disease, and decreased calorie intake [2, 3]. Empagliflozin may reach a peak plasma concentration 1.33–3.0 hours after administering the drug and the pharmacological effects may persist for more than 10 days which is evident by the existence of glucosuria after 10 days of stopping the drug [6, 7].

A keto diet is a diet where there is a great emphasis on fat consumption, low carbohydrate, and sufficient protein levels to force the body into a ketotic state as the glucose levels are decreasing [20]. Furthermore, a study assessed the short-term safety of the keto diet in type II diabetics and it revealed that a very low calorie ketogenic diet is more effective than a hypocaloric diet in terms of reducing body weight and improving glycemic control [21].

When the Adverse Drug Reaction Probability Scale, which is also known as Naranjo Scale, was used to assess the probability of the relationship between empagliflozin and EDKA in the case, the result was a total score of 6. This means that there is a probable adverse drug reaction from empagliflozin which caused euglycemic diabetic ketoacidosis [22]. The adverse event appeared after the drug was given, approved after the drug was discontinued, no alternative causes could have caused the adverse reaction, and the reaction was confirmed by objective evidence.

No case report was found of a patient who developed EDKA following the start of keto diet for three days duration and stopped all of their oral medication for 2 days. Multiple case reports discussed the development of EDKA in patients who are on a SGLT2 inhibitor after introducing a ketogenic diet for at least one week [13–16]. In our case, the patient's normal level of lactic acid rules out lactic acidosis as a side effect of metformin use as a cause of DKA. Therefore, we presume that the patient developed euglycemic DKA after introducing a keto diet to his lifestyle, which with the use of empagliflozin caused the patient to enter into euglycemic DKA. SGLT2 inhibitors can cause glucosuria up to 9–10 days after stopping the treatment [7]. The best time to stop and restart SGLT inhibitor is not known; however, it is recommended to stop it for at least 9–10 days [7]. It is viable to either restart SGLT2 inhibitors after euglycemic DKA or prescribe other antidiabetic medications [4]. Most cases in the literature discontinue SGLT2 inhibitors indefinitely after an episode of SGLT2 induces EDKA, although there is no clear guideline on whether it is best to discontinue or continue on using SGLT inhibitors [1, 2, 12, 15]. Our patient was restarted on another SGLT2 inhibitor, canagliflozin, and did not develop a recurrence of EDKA.

This case presents an important risk factor to watch for in patients using SGLT2 inhibitors. The case uniqueness is expressed by the fact that the patient did not develop euglycemic DKA until a keto diet was introduced despite discontinuing SGLT2I. SGLT2 inhibitors can stay in the system even following discontinuation of the medication. It is imperative to have the type of diet discussed with the patient who has diabetes mellitus. Furthermore, physicians should advise patients to remain hydrated and to consider stopping SGLT2 inhibitors in periods where prolonged fasting is expected or during illness.

## 4. Conclusion

This is the first case to report EDKA despite stopping SGLT2 inhibitors while being on a ketogenic diet Before starting SGLT2 inhibitors, relative contraindications and when to initiate a ketogenic diet should be discussed with the patient. This case also educates healthcare providers that EDKA can be precipitated even following the discontinuation of SGLT2 inhibitors.

## Figures and Tables

**Table 1:** Investigations.

Investigation	Result	Normal value
Serum glucose	135 mg/dl 7.5 mmol/L	70–110 mg/dl 3.9–6.1 mmol/L
HbA1c	9.7%	4.4%–5.6%
Serum sodium	132 mmol/L	136–145 mmol/L
Serum chloride	97 mmol/L	98–107 mmol/L
Serum potassium	5.5 mmol/L	3.2–5.5 mmol/L
Venous bicarbonate	6mmol/L	22–26 mmol/L
Total bilirubin	4.5 micromol/L	<21 micromol/L
Direct bilirubin	2.7 micromol/L	<5 micromol/L
AST	6 IU/L	<40 U/L
ALT	19 IU/L	<41 IU/L
ALP	139 IU/L	4–129 IU/L
Lipase	24 IU/L	13–60 IU/L
eGFR	58 ml/min/1.73m2	>60 ml/min/1.73m2
Urinary ketones	15 mmol/L (3+)	Negative
Urinary glucose	56 (4+) mmol/L	Negative
Serum lactic acid	0.8 mmol/L	0.5–2.2 mmol/L
Serum beta-hydroxybutaric acid	4.21 mmol/L	0.02–0.27 mmol/L

## Data Availability

Data sharing is not applicable to this article as no datasets were generated or analyzed during the current study.
